# Charge Sharing and Charge Loss in High-Flux Capable Pixelated CdZnTe Detectors

**DOI:** 10.3390/s21093260

**Published:** 2021-05-08

**Authors:** Kjell A. L. Koch-Mehrin, Sarah L. Bugby, John E. Lees, Matthew C. Veale, Matthew D. Wilson

**Affiliations:** 1 Space Research Centre, Department of Physics & Astronomy, University of Leicester, Leicester LE1 7RH, UK; lee@leicester.ac.uk; 2 Centre for Imaging Science, Department of Physics, Loughborough University, Loughborough LE11 3TU, UK; s.bugby@lboro.ac.uk; 3 STFC Rutherford Appleton Laboratory, Harwell Campus, Didcot OX11 0QX, UK; matthew.veale@stfc.ac.uk (M.C.V.); matt.wilson@stfc.ac.uk (M.D.W.)

**Keywords:** CdZnTe, pixel detector, X-ray detector, charge sharing, charge loss

## Abstract

Cadmium zinc telluride (CdZnTe) detectors are known to suffer from polarization effects under high photon flux due to poor hole transport in the crystal material. This has led to the development of a high-flux capable CdZnTe material (HF-CdZnTe). Detectors with the HF-CdZnTe material have shown promising results at mitigating the onset of the polarization phenomenon, likely linked to improved crystal quality and hole carrier transport. Better hole transport will have an impact on charge collection, particularly in pixelated detector designs and thick sensors (>1 mm). In this paper, the presence of charge sharing and the magnitude of charge loss were calculated for a 2 mm thick pixelated HF-CdZnTe detector with 250 μm pixel pitch and 25 μm pixel gaps, bonded to the STFC HEXITEC ASIC. Results are compared with a CdTe detector as a reference point and supported with simulations from a Monte-Carlo detector model. Charge sharing events showed minimal charge loss in the HF-CdZnTe, resulting in a spectral resolution of 1.63 ± 0.08 keV Full Width at Half Maximum (FWHM) for bipixel charge sharing events at 59.5 keV. Depth of interaction effects were shown to influence charge loss in shared events. The performance is discussed in relation to the improved hole transport of HF-CdZnTe and comparison with simulated results provided evidence of a uniform electric field.

## 1. Introduction

Semiconductors have a long history of use in the direct detection of radiation when used as the sensor material in detector systems. Compound semiconductors provide various benefits that help overcome the limitations of traditional semiconductor-based detectors while still allowing direct detection. Cadmium telluride (CdTe), for example, has a wider electronic band-gap than both silicon and germanium, enabling radiation detection at room temperature without the need for cryogenic cooling. Its large effective atomic number (Z = 50) and high density (∼5.8 g/cm${}^{3}$) provide the photon stopping power necessary for the direct detection of hard X-rays and low energy gamma-rays. Alloying with zinc to make Cadmium zinc telluride (CdZnTe) increases the resistivity by increasing the band gap [1,2]. Additionally this increases the energy required for defect formation [3] which, in theory, allows for the growth of higher purity crystals. CdZnTe is therefore a popular choice for radiation detection at room-temperature [4] and in particular, is more suited than CdTe when producing thicker sensors (>1 mm), as the larger band gap means less thermal leakage current and the higher purity can reduce polarization issues [5]. Thicker sensors will be capable of more efficiently stopping higher energy photons [6]. This is of interest in fields such as nuclear medicine [7,8] and hard X-ray astronomy [9] where portable or low mass designs are favourable but high energy photons must still be detected. Since using CdZnTe allows for direct photon detection, and without bulky cooling equipment such as cryostats, volume and mass requirements are considerably reduced.

Pixelated detector designs are commonly used to achieve imaging, photon-counting and energy-resolving requirements for the medical and astrophysics applications of Cd(Zn)Te. The small pixel effect [10] (i.e., when the pixel pitch is small compared to the detector thickness) is often employed in detectors of these materials [10,11] to minimise spectral degradation related to their poor hole transport properties [12]. This effect changes the weighting potential in the detector such that a significant charge is only induced by charge carriers drifting close to the collecting pixel, described by the Shockley–Ramo theorem [13]. In such a design, it is possible to operate the detector to predominately measure the signal induced by the electrons. This improves spectral performance since electron mobility in Cd(Zn)Te is roughly a factor 10 times larger [12] than that of the holes, meaning less carrier trapping and better charge collection efficiency.

However, when pixelated designs are used, the onset of charge sharing can can limit spectroscopic performance [14]. Charge sharing refers to when a single absorbed incident photon induces a signal across multiple pixels. This occurs due to the charge cloud spreading over neighbouring pixels from cloud growth during drift or cloud splitting as a result of X-ray fluorescence. For small pixel array detectors, charge sharing affects the majority of events at hard X-ray energies [15,16]. As sensors become thicker, the greater absorption of high energy photons and longer carrier drift distances increase the size of the charge clouds, and will result in more charge sharing. Charge sharing causes a reduction in spectral performance since carriers drifting in inter-pixel regions suffer from poorer charge collection due to the presence of distorted electric fields [17,18]. This results in charge loss and degrades the energy resolution of charge sharing events such that in practice they are either ignored, meaning the loss of counts, or included at the cost of poorer spectral resolution. This can be a limiting factor to the thickness of a CdZnTe sensor at which the photon-counting and energy-resolving performance is sufficient for nuclear and astronomical applications, if small pixel sizes are desired for imaging.

A relatively new ‘high-flux-capable’ grade of CdZnTe material (HF-CdZnTe) [19] has been developed by Redlen Technologies to combat the long-standing issue of polarization in CdTe based detectors. The polarization effects occur because of the materials proclivity for the formation of crystal impurities and defects, which worsen charge carrier mobilities due to carrier trapping in the crystal imperfections [20]. In tests with HF-CdZnTe under irradiation by very high photon fluxes (>10${}^{6}$ ph s${}^{-1}$ mm${}^{-2}$) using intense light sources like the LCLS XFEL [21] and the ESRF synchrotron [22,23] up to photon energies of 30 keV, no polarization effects were observed. While these results first and foremost show the potential of this new material for high flux applications such as in use with X-ray free electron lasers (FELs) to study the atomic structure of samples, a further implication is that these crystals are of good quality and relatively defect-free. In an early study with a new HF-CdZnTe crystal [24], Thomas et al. calculated the carrier mobilities and found significantly improved hole transport properties (2.9 ± 1.4 × 10${}^{-4}$ cm${}^{2}$V${}^{-1}$) compared with standard CdZnTe crystals (0.2 ± 1.4 × 10${}^{-4}$ cm${}^{2}$V${}^{-1}$). If less carrier trapping occurs, particularly in distorted electric fields regions close to the read-out electrode (i.e., pixel gaps), charge loss should be less severe. This would improve the energy resolution of charge sharing events.

In this work, charge sharing in a HF-CdZnTe detector of 2 mm thickness read out with the High Energy X-ray Imaging Technology (HEXITEC) spectroscopic imaging Application Specific Integrated Circuit (ASIC) [25], was investigated. We calculated the absolute amount of charge sharing at various incoming photon energies and estimated the contribution of charge loss to the energy resolution of charge sharing events. In order to do this, we first determined if diagonally adjoined pixels are separate photon events or a result of charge sharing. Results from the same analysis but for a 1 mm thick CdTe detector also used with the HEXITEC ASIC are shown. These serve as a reference to compare performance with a typical CdTe detector used in many of the applications also intended for the HF-CdZnTe detector. Furthermore, a Monte-Carlo detector model [26] was used to complement the experimentally-determined charge sharing rates and to estimate the contribution of charge sharing in an ideal noise free scenario.

## 2. Materials and Methods

The HF-CdZnTe and CdTe detectors, the HEXITEC ASIC, and the experimental and simulated data collection methods are outlined below. The different pixel event types and the algorithm used to discriminate between them is also described.

### 2.1. The Semiconductor Detectors

#### 2.1.1. HF-CdZnTe

The HF-CdZnTe detector, fabricated by Redlen technologies [27], consists of the semiconductor material as the sensor and platinum electrodes. The sensor has a thickness of 2 mm and a collecting area of 20 mm × 20 mm. The cathode is planar and the anode is pixelated by an 80 × 80 array with a pixel pitch of 250 μm consisting of a 225 μm × 225 μm pad size and an inter-pixel spacing of 25 μm. Veale at al. [28] investigated the variation in performance across a sample of 10 of these detectors all designed to the same specifications. One of the detectors included in their study (serial number D185739) is the one used in this work. More details on the design of the HF-CdZnTe detectors and the assembly to the HEXITEC ASIC can be found in their paper.

#### 2.1.2. CdTe

The CdTe detector is manufactured by Acrorad Ltd and has the same collecting area of 20 mm × 20 mm as the HF-CdZnTe detector but a sensor thickness of 1 mm. The electrodes form blocking (Schottky) contacts with a planar platinum cathode and pixelated aluminium anode. The pixilation of the anode also consists of an 80 × 80 array with 250 μm pixel pitch, however the pad size is 200 μm × 200 μm and inter-pixel spacing is 50 μm.

The design of the CdTe detector differs from the HF-CdZnTe detector in a number of significant ways (electrode contacts, thickness and inter-pixel gap size). What affect these individual design changes have on detector performance cannot be accurately separated. However, Acrorad CdTe detectors have over recent years been used in a large number of studies, achieving good results [29,30,31]. Coupled with the HEXITEC ASIC, excellent Full Width at Half Maximum (FWHM) energy resolution for single pixel events (0.75 keV at 59.5 keV across all pixels) has been achieved as well as good pixel count uniformity [29]. This makes the CdTe detector a relevant and suitable point of reference to compare to the HF-CdZnTe detector.

### 2.2. The HEXITEC Detector System

The HEXITEC ASIC is a spectroscopic imaging read-out chip developed by the Science and Technology Facilities Council (STFC) to detect X-rays and gamma rays [25]. When a detector is coupled to the ASIC, spectroscopic observations recording the position and energy of all incident photons attenuated by the attached sensor can be performed. The HEXITEC ASIC has 6400 channels arranged in an 80 × 80 array on a 250 μm pixel pitch. Each ASIC pixel consists of a charge amplifier, shaping amplifier and a peak-track-and-hold circuit such that the induced charge is recorded as a voltage at the pixel level. The peak-track-and-hold records the maximum detected voltage in a frame. Photon pileup (see Section 2.5) will therefore only occur if two separate photon events occur within the shaping rise (2 μs) and recovery time (10 μs) [32]. The magnitude of the recorded voltage is proportional to the energy of the detected photon(s), converted with a per pixel energy calibration.

The HEXITEC ASIC only records the signal induced at the pixelated anode. Since the HEXITEC detector system is designed for back-illumination (i.e., through the cathode), by applying a reverse bias to the cathode, single carrier sensing of the electrons is achieved, with holes drifting to the cathode and electrons to the anode. Both detectors used for this work have a small pixel geometry and therefore also benefit from the small pixel effect. Thomas et al. [24] shows simulated one-dimensional weighting potentials for both detector geometries. The detector geometry favours the electron signal because electrons will always traverse the regions of high weighting potential nearest to the anode. Holes will only induce a significant signal if the cloud is deposited directly in the high weighting potential region, as they will then drift away from the anode towards the cathode. The hole signal is therefore not eliminated entirely in an electron sensing geometry, but it is reduced.

Observations are carried out through a data acquisition system (DAQ) [33] into which the coupled detector ASIC module is mounted. The DAQ digitizes the analogue voltage signal from the ASIC and contains the electronics to control the read-out frame rate, apply the bias voltage to the detector and monitor temperature values. An ether-net connection is used to operate the DAQ from a PC, with control through a custom software.

### 2.3. Experimental Data Collection and Calibration

#### 2.3.1. HF-CdZnTe Calibration and Data

**Figure 1:**
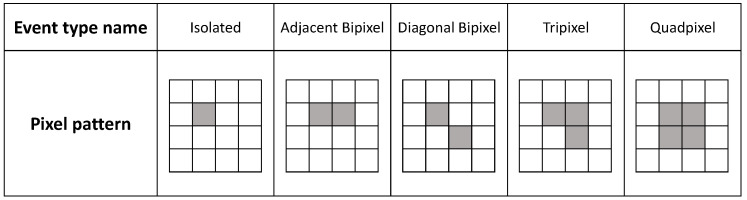
Different photon event types considered by the analysis algorithm and added to an energy spectrum. The event type is defined by the pixel pattern and number of pixels in an event.

A series of flood images using different radioactive isotope sources were taken with the HF-CdZnTe detector. The sources used for calibration and their most prominently observed energies are listed in Table 1. The ASIC frame read out was set to 1.6 kHz for each observation. An energy calibration was produced for each pixel using the photopeaks at 5.95, 22.00, 59.54 and 122.10 keV. The chosen photopeaks were all clearly resolved and contained at least a 1000 counts (∼3% uncertainty in counts) in each pixel and provided calibration across a large energy range. Only isolated events (see Figure 1) were used for the energy calibration and counts below a noise threshold of ∼3 keV were first removed.

The sources were aligned with the centre of the detector at a distance of 38 cm to ensure uniform illumination and low frame occupancy rates. The frame occupancy is defined as the percentage of pixels in a frame recording a count above the noise threshold. At the low frame occupancy rates in each observation with the HF-CdZnTe detector (listed in Table 1), the likelihood of multi-pixel events occurring due to photon pile-up rather than charge sharing can be assumed to be very low. This was an important requirement for accurately calculating the charge sharing proportions in the detector. Although not all peaks in Table 1 were used for calibration, all were used for the charge sharing analysis as a function of energy (Section 3.3) and are therefore listed.

The energy calibration method used is described in detail by Scuffham et al. [34]. A linear fit giving the relationship between the digitised energy in ADU (analogue-to-digital unit) and energy in keV was obtained for every pixel from the calibration photopeaks. This fit was used to convert and calibrate ADU energies to keV on a per pixel basis. The fit parameters and fit statistics were also used to determine any poorly performing pixels. Pixels for which the linearity ($R^{2}$ value), intercept or slope deviated by more than 5σ from the average value of all pixels, were excluded. In total this lead to the removal of 83 pixels from analysis, 80 of which were along a single column at the edge of the detector.

#### 2.3.2. Frame Occupancy Measurements with the HF-CdZnTe Detector

Using the HF-CdZnTe detector, additional flood images were taken with the ${}^{241}$Am source in order collect data with different frame occupancy rates. Both the distance of the source and the ASIC frame read-out rate were varied to influence the frame occupancy, shown in Table 2. These data were used to investigate the best event reconstruction method for diagonally adjoined bipixel events (Section 3.1).

#### 2.3.3. DAQ Operation for the HF-CdZnTe Detector

For all measurements with the HF-CdZnTe detector, the HEXITEC DAQ was operated with a bias voltage of −750 V (375 V mm${}^{-1}$) applied to the detector cathode and the detector temperature was maintained at 28 ±1 ${}^{\circ }$C. For Schottky contact CdTe detectors, it is necessary to periodically refresh the bias to avoid bias induced polarization [35]. For the HF-CdZnTe detector, differences in the contact technology result in less charge build up across the detector, such that it was not necessary to periodically refresh the bias.

#### 2.3.4. CdTe Data

The CdTe detector data and calibration is from Bugby et al. [36]—which used the exact DAQ and same type of HEXITEC ASIC as for the HF-CdZnTe measurements. Flood images were taken with the same sources listed in Table 1, providing data at the same photopeak energies. In a previous study [26], additional data was collected with the CdTe detector using an X-ray tube which provided data at three more peaks (32.5, 38.0 and 45.8 keV). Frame occupancy rates for all the CdTe detector data was kept below 1%. The detector was operated at a bias of −500 V (500 V mm${}^{-1}$) and the temperature remained at 28 ± 1 ${}^{\circ }$C. To prevent the known time-dependent bias induced polarization effects of Schottky contact CdTe [35], the bias voltage was periodically reset every 60 s by switching the bias to 0 V for 2 s.

### 2.4. Simulated Data from Monte Carlo Modelling

A Monte-Carlo model has been developed to simulate the spectroscopic performance in pixelated CdTe detectors and has shown good agreement with experiment [26]. This model has been extended to work with CdZnTe and was used in this study to simulate the response of a 2 mm CdZnTe detector to various photon energies covering the range given by the photopeaks in Table 1.

For each simulation, 10${}^{7}$ input photons were used. The detector temperature was set to 28 ${}^{\circ }$C and the bias voltage to −750 V. The pixel size in the model was set to the electrode pad size of 225 μm. The simulated data were analysed using the same event reconstruction algorithm as for the experimental data (described in Section 2.5), using a noise threshold of 3 keV and spectral bin size of 0.3 keV.

### 2.5. Photon Event Types and Event Reconstruction

Photon counts recorded by the read-out ASIC can appear in many different pixel patterns or, as we refer to them here, as different ‘event types’. The number of pixels an absorbed photon induces a charge in (its multiplicity), and their arrangement, determines the event type. If all of the charge is contained within a single pixel, it is an isolated event. If the charge is collected over multiple neighbouring pixels, it is a shared (charge sharing) event (e.g., bipixel, tripixel, quadpixel). Figure 1 shows a schematic of each event type considered in this work. Events with multiplicity >4 have a very low incidence rate (∼0.1% at 59.5 keV) for the photon energies considered in this work and are therefore ignored.

Excluding pile up, a shared event can occur either from a single charge cloud inducing charge over multiple pixels, or due to X-ray fluorescence. The self fluorescence in materials containing high-Z elements such as Cd and Te have energies large enough that their mean attenuation ranges (60– 100 μm) can be comparable to the pixel size. If fluorescence photons are generated due to the photoelectric absorption of the incoming photon, there is a high chance of the fluorescence escaping the incident pixel and stopping in an adjacent pixel, resulting in a fluorescence shared event. For the CdTe detector, at an incoming photon energy of 59.5 keV, ∼12% of all adjacent bipixels and ∼65% of all tripixels contain a shared fluorescence photon [26]. It is useful to distinguish between shared events that result from a single cloud centre and those due to fluorescence as their energy response can be quite different—with fluorescence shared events typically showing better energy resolution as they are less affected by charge loss [37].

An event analysis algorithm was used in post-processing of the experimental and simulated data to identify photon counts belonging to any of the event types shown in Figure 1. Pixels that are in one of the arrangements shown in Figure 1 are considered as part of the same incoming photon event and their energies summed. Different pixel patterns of the same number of pixels such as linear tri- or quadpixels were ignored by the algorithm as their incidence rate is very low (∼0.3% at 59.5 keV) and they are more likely the result of photon pileup.

Diagonal bipixels were observed to have a slightly larger incidence rate of 1.5% at 59.5 keV and are expected to be created from fluorescence. Diagonal bipixels are therefore considered by the algorithm. However, since photon pileup creates events which appear the same as a shared event (but are in fact due to signals in neighbouring pixels from multiple distinct photons instead of a single photon), diagonal bipixels may be dominated by pileup. Section 3.1 discusses the best reconstruction method for diagonal bipixels.

A detector will record energies that are not the result of excited charges from an absorbed photon but instead thermal excitations in the semiconductor sensor. These count are typically of low energy and are regarded as noise. By using a low energy threshold this noise can largely be removed. The event analysis algorithm applies an energy threshold to each pixel before determining the event type.

For each event type separately, an energy spectrum was produced by binning the reconstructed energies into 0.3 keV bins. Unless stated otherwise, a 3 keV noise threshold was applied to each pixel.

## 3. Results

### 3.1. Diagonal Bipixels

Diagonally adjoined pixels are commonly treated as separate events or deemed as erroneous events and ignored entirely because the charge from a single charge cloud is physically unlikely to produce a diagonal pattern. This is because charge clouds are understood to take a spherical or ellipsoidal form through thermal diffusion and electron repulsion during drift [38]. Diagonal patterns are therefore assumed to be the result of photon pileup or detector noise. However, at incoming photon energies above the K-shell binding energy in high-Z detectors, diagonally adjoined events can be the result of a fluorescence photon escaping into a diagonally adjacent pixel as fluorescence is randomly emitted over all solid angles. In this case, the energies in the diagonally adjoined pixels should be combined in order to fully recover the energy of the incoming photon. For larger multi-pixel event types, the number of pixel pattern variations, particularly when including diagonally adjoined pixels, increases rapidly. Therefore, we have restricted the analysis of diagonally adjoined events to bipixels only.

#### 3.1.1. Energy Response

**Figure 2:**
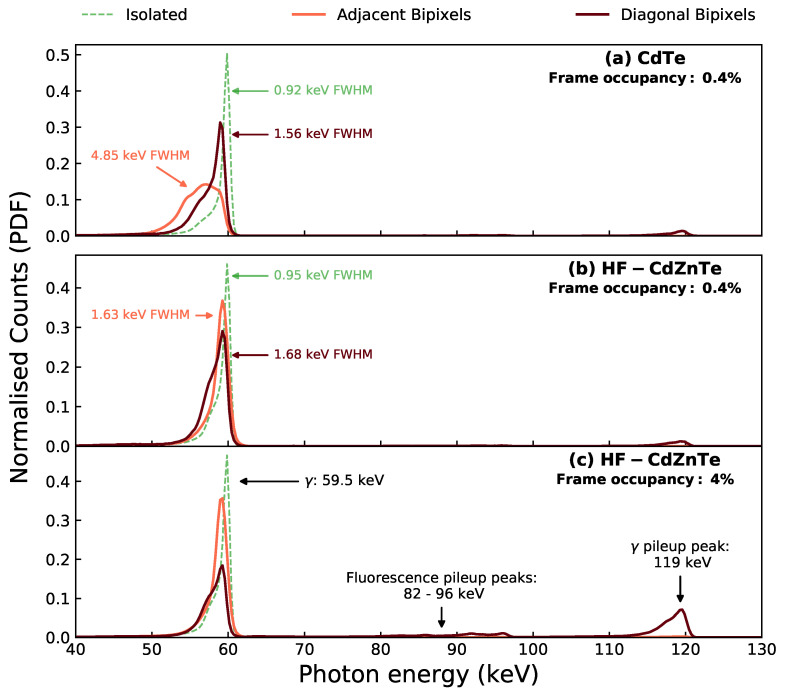
The whole detector (all pixels) ${}^{241}$Am spectra for isolated events, adjacent bipixels and diagonal bipixels. The spectra are all given as probability density functions, obtained by normalisation of the total number of counts and bin size (0.3 keV). (**a**) the data collected with the CdTe detector at a frame occupancy of 0.4%. (**b**) the data collected with HF-CdZnTe detector with the source at 38 cm, giving a frame occupancy of 0.4%. (**c**) the data collected with HF-CdZnTe detector with the source at 13.5 cm, giving a frame occupancy of 4%.

Figure 2 shows the energy spectrum as a probability density function (PDF) for the diagonal bipixels, adjacent bipixels and isolated events from ${}^{241}$Am observations with both detectors. For the HF-CdZnTe detector, the event type spectra are shown for two observations of different frame occupancy—0.4% (Figure 2b) and 4% (Figure 2c). The lower frame occupancy gives a direct comparison to the CdTe observation of the same frame occupancy shown in Figure 2a. The 4% frame occupancy data serves to show how the recorded energy response of the diagonal bipixels changes with frame occupancy.

**Figure 3:**
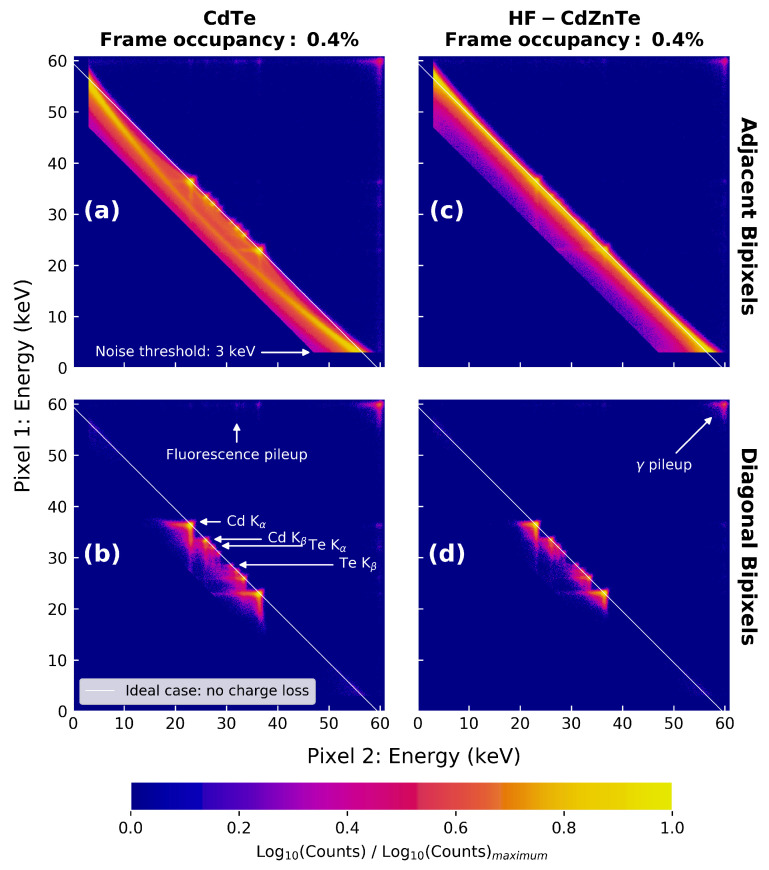
Two-dimensional distributions of the energy split between the two pixels in adjacent and diagonal bipixel events for the ${}^{241}$Am data with 0.4% frame occupancy. The panels are: (**a**) CdTe data for adjacent bipixels; (**b**) CdTe data for diagonal bipixels; (**c**) HF-CdZnTe data for adjacent bipixels; (**d**) HF-CdZnTe data for diagonal bipixels. An Energy window from 50 to 130 keV is applied to each distribution. Annotations highlighting specific events apply to all images but are only shown once where they are clearly visible. The normalised log${}_{10}$ of the counts are shown, with each panel normalised by its own distribution’s maximum bin counts.

The differences in energy response between the event types and detectors is also shown by the analysis presented in Figure 3. Figure 3 uses 2-dimensional distributions to show the energy split of the 59.5 keV peak between the two pixels in the bipixel event. This is shown for the ${}^{241}$Am data with 0.4% frame occupancy for the CdTe (Figure 3a,b) and HF-CdZnTe detector (Figure 3c,d). The energy split analysis reveals if the energy across bipixel events is fully recovered or if instead charge loss is occurring. The diagonal path through the middle of the distribution, shown by the solid white line, indicates an energy split across the two pixels that always adds up to the photopeak energy of 59.5 keV. Counts on this line therefore do not show any charge loss, whereas counts deviating below the line do.

Within a ±10% window of the 59.5 keV photopeak in Figure 2a, ∼3.8% of all bipixels (1.5% of all events) were found to be diagonal bipixels. The diagonal energy split distributions in Figure 3 reveal that these events are a consequence of fluorescence. The majority of counts are concentrated in bright spots where one of the pixels contains the energy of a K-shell fluorescence photon from either Cd or Te (labelled in Figure 3c), and the second pixel contains the escape peak energy. Since the diagonal bipixels are formed by fluorescence, they are not expected to suffer from charge loss. This is supported by their energy split distributions, which show the bright fluorescence spots positioned along the diagonal path of complete charge collection.

The adjacent bipixel energy split distributions (Figure 3a,c) show counts at all energy pairs, in addition to the fluorescence bright spots. Adjacent bipixels are therefore the result of either fluorescence or charge spreading events. In the case of the CdTe detector, the energy split distribution shows a curve which significantly deviates from the solid white line. Counts along this curve are the charge spreading events which suffer from charge loss due to incomplete charge collection from charges drifting in the inter-pixel gap. For the HF-CdZnTe detector (Figure 3c), this curve follows the solid white line more closely—indicating less charge loss. Consequently, the energy resolution of the adjacent bipixel photopeak in the HF-CdZnTe detector is significantly better at 1.63 keV FWHM (Figure 2b) compared with 4.85 keV FWHM for the CdTe detector (Figure 2a).

The energy resolution of the diagonal bipixel photopeaks are more comparable between the two detectors, as charge loss is not a factor in their response. For the CdTe detector, which exhibits large amounts of charge loss, including diagonal bipixels has, in addition to recovering more counts, the further benefit of recovering those counts at a much better energy resolution (Figure 2a).

The frame occupancy of an observation also has an impact on the spectral response because the probability of photon pileup increases. The spectra in Figure 2c show that the diagonal bipixel response is more susceptible to pileup than the adjacent bipixel response. The γ pileup peak at 119 keV, which consists of two distinct 59.5 keV photons, contains ∼25% of all diagonal bipixels at just 4% frame occupancy. For comparison, only about 1% of all adjacent bipixels are in the γ pileup peak. Some photon pileup also occurs with the incoming photon energy of 59.5 keV in one pixel, and a Cd or Te K-shell fluorescence photon or escape energy in the second pixel. These we refer to as the fluorescence pileup peaks, labelled in Figure 2c. The γ and fluorescence pileup counts are also visible in the energy split distributions (labelled in Figure 3).

#### 3.1.2. Event Reconstruction Method

**Figure 4:**
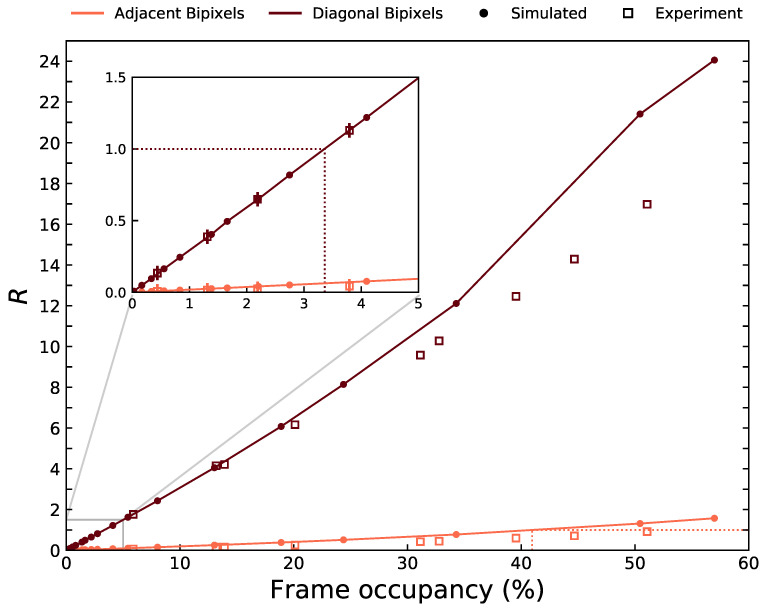
Equation (1) (*R*) as a function of frame occupancy for the experimental observations from Table 2 and simulated data. The filled circles are the result from simulated data and the empty squares from experiment. The solid lines connect the simulated values. Different colours are used to distinguish the event types. Error are estimated at R±0.05 determined by the change when using ±15% energy windows.

As the distribution of diagonal bipixels changes with event occupancy, it is necessary to adjust the reconstruction parameters used in order to recover the maximum number of counts at the correct photopeak energy. We define a ratio, *R*, as the number of counts recovered at the correct peak energy when treating diagonal bipixels as two isolated events, over the number of counts recovered at the correct energy when treating diagonal bipixels as a shared event. The number of counts for either of these cases can be extracted from the peaks in the diagonal bipixel spectrum (seen clearly in Figure 2c), such that *R* is defined as

(1) $$R=\frac{(\mathrm{Counts}\;\mathrm{in}\;\gamma \;\mathrm{pileup}\;\mathrm{peak})\;\times \;2\;+\;\mathrm{Counts}\;\mathrm{in}\;\mathrm{fluorescence}\;\mathrm{pileup}\;\mathrm{peaks}}{\mathrm{Counts}\;\mathrm{in}\;\mathrm{primary}\;\gamma \;\mathrm{photopeak}}$$

with energy windows of ±10% used around each peak. If R<1, the energy in the two diagonally adjoined pixels should be summed (i.e., shared events are assumed) in order to recover more counts at the correct energy. Whereas if R>1, diagonal bipixels should be treated as two isolated events. *R* was calculated for HF-CdZnTe data at different frame occupancies. For comparison, *R* was also calculated for adjacent bipixels. *R* was also calculated from simulated data from the Monte-Carlo detector model. Figure 4 shows the value of *R* calculated as a function of frame occupancy for both bipixel types.

At a frame occupancy of 3.4%, R=1 for the diagonal bipixels. This suggests that below a frame occupancy of 3.4%, more counts will be recovered at their correct energy by treating diagonal bipixels as shared events instead of isolated events. Above this frame occupancy, diagonal pixels should be regarded as photon pileup events and consequently be treated as two isolated events. The fraction of adjacent bipixels due to photon pileup is much lower. Even at the highest frame occupancy obtained experimentally (51%), R=0.91 for the adjacent bipixels. The model slightly overestimates the number of piled-up adjacent bipixels at all frame occupancy rates—predicting R=1 at a frame occupancy of 41%. For the diagonal bipixels, the simulated results from the model show excellent agreement to experiment up to a frame occupancy of 15%. Simulations from the detector model can therefore be used to inform on the frame occupancy threshold at which the treatment of diagonal bipixels should be altered. This could prove useful for detectors of different design, particularly pixel size, in order to recover as many counts as possible.

The frame occupancy is the ideal metric to use as it is determined directly from the data with no prior information such as pixel size or frame rate needed. The choice of the diagonal event reconstruction method can therefore be adopted automatically during an acquisition if required, with the choice of the threshold *R* chosen based on the statistical requirements of the application. Although *R* has been determined here from analysis using only monochromatic sources, the results can also be applied to polychromatic sources if the majority of emitted counts are above the detector crystal absorption edges.

### 3.2. Energy Resolution and Charge Loss of Multi-Pixel Events

The ability of a detector to spectrally resolve photons of different energy is measured by its energy resolution. Energy resolution can be quantified as the full width at half maximum (FWHM) of a photopeak at a specific energy. A number of factors influence the energy resolution which together determine the width of the recorded photopeak. The dominant factors for compound semiconductors are statistical fluctuations in carrier generation (or Fano noise), electronic noise, and incomplete charge collection. Fano noise stems from the variance in electron-hole pair creation across the band-gap, whereas electronic noise is due to thermal charge leakage (dark current) and noise from the DAQ and ASIC read-out process. Incomplete charge collection includes carrier trapping and charge loss to the inter-pixel gap. In this section, by calculating the FWHM energy resolutions of the different event types, we separate and determine the magnitude of the charge loss component only (from its contribution to photopeak broadening) for the multi-pixel events of both detectors.

#### 3.2.1. Event Type Spectra

**Figure 5:**
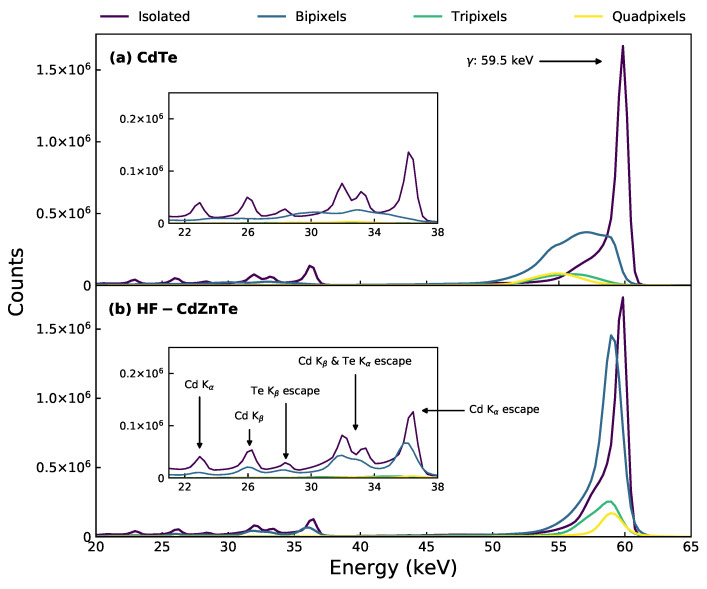
Event type spectra for the whole detector (all non calibration excluded pixels) ${}^{241}$Am observation with the (**a**) CdTe detector and (**b**) HF-CdZnTe detector. The bipixel spectrum includes both the adjacent and diagonal bipixels. Inset plots are a magnification of the same spectra at the fluorescence and escape peak energies (21–38 keV). Annotations in the figure apply to both (**a**) and (**b**).

Figure 5 shows the different event type spectra for the ${}^{241}$Am observation taken with the CdTe (a) and HF-CdZnTe (b) detectors. The spectra include counts from all detector pixels, except those removed during calibration. The primary γ photopeak at 59.5 keV is resolved in all of the event type spectra by both detectors. The escape peaks and fluorescence peaks are clearly resolved in the isolated spectrum for both detectors, but are only resolved in the bipixel spectrum for the HF-CdZnTe detector. The HF-CdZnTe detector shows a clear improvement in energy resolution for all multi-pixel event types when compared to the CdTe detector.

Since the fano- and electronic noise component are similar in the two detectors (see Section 3.2.3), the better energy resolution of the multi-pixel events indicates less charge loss is occurring in the HF-CdZnTe detector. The low energy tails seen on the LHS of the 59.5 keV photopeaks are due to carrier trapping [39] (resulting in so-called hole tailing) and the noise threshold.

#### 3.2.2. Energy Resolution Calculations

The FWHM energy resolution (ΔE) was calculated for the isolated, adjacent bipixel and quadpixel event photopeaks at energies 14.4, 22.0, 59.5, 88.0, 122.1 and 140.5 keV. This was done by fitting a Lorentzian function to the counts in the photopeak in question. Energy windows of ±5 keV were used around the photopeaks during fitting in all cases except for the CdTe multi-pixel photopeaks for which +5/−10 keV windows were applied (due to the wider peaks). A Lorentzian function was used (instead of a Gaussian) to account for the low energy tail, minimising the influence of the tail on the measured photopeak width. This helps ensure that the measured photopeak width is not dependent on the applied noise threshold. The photopeaks contained the counts from all detector pixels (except those removed during calibration).

Diagonal bipixels were not included as they have been shown to be due to fluorescence only and therefore do not exhibit charge loss. Tripixels were not included in this analysis for the same reason, as they are primarily the result of fluorescence [26].

**Figure 6:**
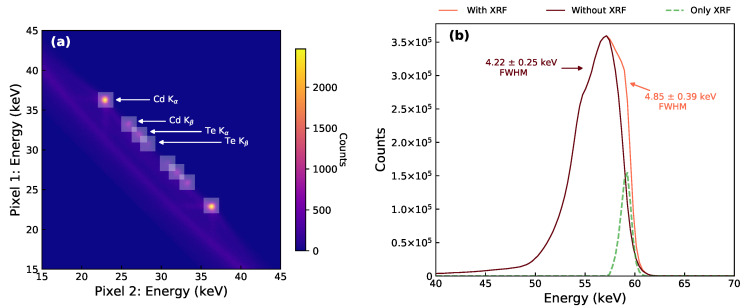
Plots contain data of only adjacent bipixels from the ${}^{241}$Am observation with the CdTe detector, within an energy window of 40–70 keV. (**a**) Energy split distribution from Figure 3a (magnified around the fluorescence spots) show the positions of the 2D energy windows applied in order to remove the fluorescence events. (**b**) The adjacent bipixel photopeak before and after removing the fluorescence events within the 2D energy windows in (**a**), and a peak containing only the energy windows fluorescence events. XRF refers to the X-ray fluorescence events.

For the CdTe adjacent bipixel photopeaks, at energies above the Cd K-edge, the fluorescence events were removed before fitting the Lorentzian function. This was done because the fluorescence events have a very different response compared with the charge spreading events, with peak positions at 58.95 and 57.15 keV, respectively, (Figure 2a). The fluorescence events were identified and removed by applying 2-dimensional (2D) energy windows (i.e., an energy window to each pixel in the event) to the adjacent bipixels. Figure 6a is a magnification of the ${}^{241}$Am bipixel energy split distribution from Figure 3a and shows the energy positions of the 2D windows applied around the fluorescence counts. Figure 6b shows the 59.5 keV adjacent bipixel photopeak before and after removing the fluorescence counts, as well as a peak containing only the fluorescence adjacent bipixels within the 2D energy windows. The figure shows that removing the fluorescence events has a significant effect on the measured FWHM. The optimum energy window width, at which the maximum amount of fluorescence events were removed without removing charge spreading events, was determined to be 1.8 keV. This was the largest energy window before counts in the energy bin containing the maximum number of counts for the charge spreading peak (57.0–57.3 keV) began to decrease. Fluorescence events were not removed from the HF-CdZnTe adjacent bipixels as this could not be done without also removing many charge spreading events (Figure 3c). However, since the response of the fluorescence and charge spreading events is more similar in the HF-CdZnTe detector (Figure 2c), it is expected the fluorescence events will have less of an affect on the measured FWHM.

**Figure 7:**
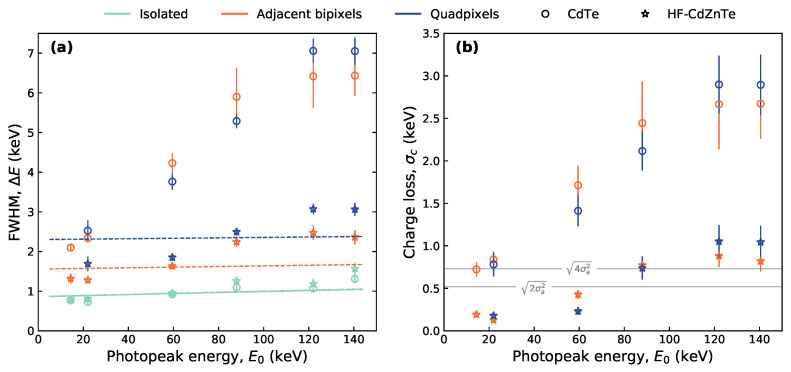
(**a**) FWHM energy resolution at different photopeak energies. Lines represent the fit of Equation (3) to the FWHM values—solid line for CdTe and dotted line for HF-CdZnTe. Errors on the FWHM values are the standard errors obtained from least-squares fitting of the Lorentzian function to the photopeaks. (**b**) The contribution of the charge loss component to photopeak broadening, expressed as a standard deviation of a Gaussian distribution. Errors are propagated from the errors on the FWHM and $\sigma_{a}$. The solid horizontal lines indicate the electronic noise contribution from all pixels in the event for bipixels ($\sqrt{2\sigma_{a}^{2}}$) and quadpixels ($\sqrt{4\sigma_{a}^{2}}$). For both plots, colours distinguish the event types and markers distinguish the detectors. A sufficient amount of quadpixels were not recorded at 14.4 keV—the respective value is therefore missing from both plots, for both detectors.

The resulting FWHM for each fitted photopeak for different event types in both detectors are plotted in Figure 7a. Errors for some photopeak FWHM values, particularly for the CdTe multi-pixel event peaks, are relatively large. This is due to charge loss complicating the peak shapes away from standard distributions such as Lorentzian or Gaussian.

#### 3.2.3. Charge Loss Calculations

Analytically, the broadening of a photopeak is typically expected to follow a Gaussian distribution with a standard deviation, σ, given by the sum of the individual broadening components [12],

(2) $$\sigma =\sqrt{F\omega E_{0}+m\sigma_{a}^{2}+\sigma_{c}^{2}},$$

where the first term is the Fano noise consisting of the Fano factor, *F*, the electron-hole pair creation energy, ω (4.43 eV and 4.64 eV in CdTe and CdZnTe, respectively, [12]) and the photopeak energy, $E_{0}$. The second and third terms are the variance in peak width due to electronic noise, $m\sigma_{a}^{2}$, where *m* is the event multiplicity, and the variance in peak width due to incomplete charge collection, $\sigma_{c}^{2}$. The reported Fano factor values for CdTe and CdZnTe range from 0.06 to 0.14 [6]—we use a value of 0.1 for both materials, as is generally done [39]. The energy resolution, ΔE, can then be expressed as the FWHM of a Gaussian distribution (2.355σ) with standard deviation from Equation (2),

(3) $$\Delta E=2.355\sqrt{F\omega E_{0}+m\sigma_{a}^{2}+\sigma_{c}^{2}}.$$

Typically Equation (3) will not include the event multiplicity term *m*. However, since we calculate the energy resolution for multi-pixel events, and each pixel in the HEXITEC ASIC contains its own read out circuit, the electronic noise contribution from each individual pixel in the event must be considered.

For each event type in Figure 7a, Equation (3) was fit to the calculated FWHM values as a function of photopeak energy $E_{0}$. The fitted FWHM relationships are shown in Figure 7a by the solid (CdTe) and dotted (HF-CdZnTe) lines. For isolated events (m=1), we assumed the energy resolution is not affected by charge loss and therefore set $\sigma_{c}=0$. From the isolated events fit, the peak broadening due to the electronic noise from a single pixel ($\sigma_{a}$) was determined. The value for $\sigma_{a}$ for the CdTe and HF-CdZnTe detector are shown in Table 3. The bipixel and quadpixel FWHM values were fit by setting *m* to the multiplicity of the event and fixing $\sigma_{a}$ to the value determined from the isolated events fit. A fit could not be found to the CdTe detector FWHM values for both the bipixel and quadpixel photopeaks due to the significant increase of the FWHM values with energy. For the HF-CdZnTe detector, fits were achieved with the $\sigma_{c}$ values shown in Table 3. The fits however are poor because the FWHM values, as for the CdTe detector, increase at a faster rate with energy than is predicted by Equation (2). The source of this discrepancy is believed to be related to the depth of interaction with photon energy and the poor hole transport—discussed in Section 3.2.4.

Since good fits could not be obtained by fitting Equation (2) to all FWHM values for the multi-pixel event types, $\sigma_{c}$ was calculated separately at each energy. This was done using Equation (2) with the $\sigma_{a}$ value in Table 3 for the respective detector, and *m* equal to the multiplicity of the event. The values for $\sigma_{c}$ determined this way are shown in Figure 7b. For comparison, Figure 7b also shows the magnitude of the electronic noise broadening component from two and four pixels (solid horizontal lines).

Both detectors show excellent energy resolution for isolated event photopeaks, with the HF-CdZnTe performing nearly identically to the CdTe despite being twice as thick (2 mm/1 mm) and with a weaker applied electric field (375 Vmm${}^{-1}$/500 Vmm${}^{-1}$). Within errors, the contribution of electronic noise to photopeak broadening ($\sigma_{a}$) was found to be the same for the two detectors (Table 3).

In the HF-CdZnTe detector, the broadening of the photopeaks due to charge loss is less significant than the electronic noise contribution from all pixels in the event. This applies up to energies of at least 60 keV, for both bipixels and quadpixels. At 59.5 keV, by considering only the electronic noise contribution per pixel (i.e., $\sigma_{c}=0$) under the experimental conditions, the best achievable energy resolution for bipixels is 1.30 ± 0.11 keV FWHM. The FWHM of the observed bipixel peak by the HF-CdZnTe detector is not far from this value at 1.63 ± 0.08 keV—a significant improvement over the CdTe detector (4.22 ± 0.08 keV FHWM).

#### 3.2.4. Depth of Interaction and Charge Loss Correlation

The amount of charge loss was shown to vary with photon energy (Figure 7b). For this reason, the FWHM values of the multi-pixel event photopeaks (Figure 7a) could not be described with the analytical equation for detector energy resolution (Equation (3)). The variation of $\sigma_{c}$ with energy is thought to be correlated with the photon depth of interaction due to the poor hole transport typically reported for Cd(Zn)Te detectors [12,40].

**Figure 8:**
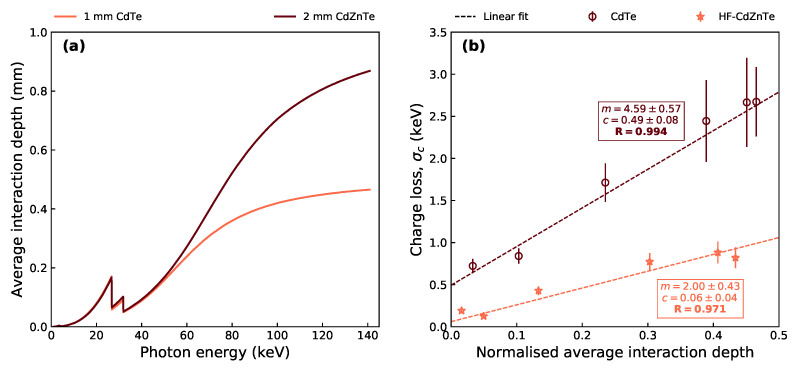
(**a**) Average interaction depth for photons of different energy in a 1 mm and 2 mm block of CdTe and CdZnTe, respectively. Each value ranging from 1 to 141 keV in steps of 0.1 keV was calculated using simulated data from the detector model, which determined the average attenuation depth from the 10${}^{7}$ incident photons which were attenuated within the crystal block. Attenuation coefficients from the NIST XCOM database were used [41]. (**b**) Correlation plot of the charge loss contribution to photopeak broadening for adjacent bipixels, against the average interaction depth of the photon energy at which $\sigma_{c}$ was measured. The interaction depth is normalised by the detector thickness. *R* is the Pearson correlation coefficient from the linear fits, while *m* and *c* are the slope and y-axis intercept, respectively.

Figure 8a shows the average photon interaction (i.e., attenuation) depth with photon energy—simulated for a 1 mm and 2 mm thick block of CdTe and CdZnTe material, respectively. For a detector of finite thickness, the average depth at which photons are attenuated will level off and plateau at half the detector thickness. This levelling-off is observed in the measured FWHM and $\sigma_{c}$ values for the multi-pixel events in both detectors (Figure 7). A similar trend with energy has been observed by Bugby et al. [36] in calculations of a charge loss correction parameter.

For smaller photon energies, the average depth of interaction is much further from the anode than at larger photon energies. Due to the small-pixel effect in these detectors, only a very small portion of the induced charge will come from holes drifting far from the anode. Therefore, at lower energies, $\sigma_{c}$ is smaller because the poor hole transport has less of an impact on the energy resolution of shared events than at larger energies.

Using the relationship between photon energy and average interaction depth from Figure 8a, the $\sigma_{c}$ values for the adjacent bipixels from Figure 7b were plotted against their equivalent average interaction depths (Figure 8b). The quadpixels were omitted for clarity and because they lack the data point from the 14.4 keV photopeak. The interaction depths were normalised by the detector thickness for better comparison between the CdTe and HF-CdZnTe. Linear fits were performed to determine the correlation between the two parameters. The results show a strong correlation between $\sigma_{c}$ and the photon interaction depth for both detectors, with Pearson’s correlation coefficient (*R*) equal to 0.994 and 0.971 for the CdTe and HF-CdZnTe values, respectively. However, given the large errors on $\sigma_{c}$ and a limited number of data points, it is possible the correlation is not linear and other effects influencing the trend of $\sigma_{c}$ cannot be ruled out.

The reduced charge loss in the HF-CdZnTe detector will predominately be due to the smaller 25 μm pixel gaps (see Section 4). The correlation in Figure 8b, however, suggests that the hole transport influences the amount of charge loss that occurs. For photons absorbed immediately below the cathode (i.e.; at an interaction depth of zero), the charge loss can be assumed to be primarily due to the inter-pixel spacing (and electron transport) rather than the hole transport. As expected, at zero depth, $\sigma_{c}$ is greater in the CdTe detector ( 50 μm pixel gaps) than in the HF-CdZnTe detector (shown by the intercept values from the fits in Figure 8b). The slope for the HF-CdZnTe fit is smaller than for the CdTe fit. This could be due to the improved hole transport, but is likely also related to the effective electric field across the detector, which is thought to be more uniform in the HF-CdZnTe detector (see Section 3.3).

### 3.3. Charge Sharing Proportions

The proportion of detected photons that were recorded as shared events was calculated at each photopeak energy in Table 1. This was done for both the experimentally collected HF-CdZnTe data and simulated CdZnTe data. Counts were considered to belong to a photopeak if they were within a ±10% energy window of the photopeak in question. The shared events proportion was then determined by summing all counts belonging to any of the multi-pixel event types (from Figure 1) and normalising by the total number of all event type counts within the energy window.

**Figure 9:**
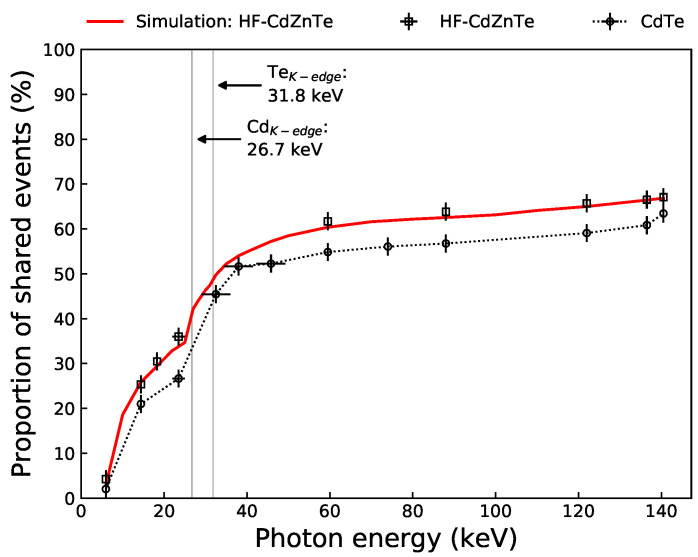
Total charge sharing proportion including all multi-pixel events as a function of photon energy—for the HF-CdZnTe detector data (squares), CdTe detector data (circles) and simulated data (solid red line). The HF-CdZnTe charge sharing proportions were calculated at each photopeak energy in Table 1. The 22 and 24.9 keV photopeaks were combined because they could not be resolved for the tripixel and quadpixel events. Taking the relative incidence of the two combined peaks into account, the line energy was plotted at 23.10 ± 1.00 keV. Errors are estimated at ≤2% determined by the change in percentage when varying the energy window from ±10% to ±15% when possible. The CdTe results are from [26,36] after re-analysis to include diagonal bipixels—joined by a dotted line to help visualisation.

Figure 9 shows the proportion of shared events as a function of photon energy for the experimental and simulated HF-CdZnTe data. The charge sharing proportions for the CdTe detector, originally calculated in [26,36], are also shown (after re-analysis of the data to include diagonal bipixels above the K-shell absorption edge energies). As is expected because of the larger thickness and smaller inter-pixel gaps, the proportion of events exhibiting charge sharing in the HF-CdZnTe detector is slightly greater than in the CdTe detector. At 59.5 keV, 61.6% of all events are shared in the HF-CdZnTe detector compared with 54.8% in the CdTe detector.

The change in the proportion of shared events with photon energy depends on a number of factors. The main ones are the geometry of the detector, charge cloud size (i.e., photon energy), the noise threshold and X-ray fluorescence from the sensor material. At low photon energies, the number of shared events is heavily suppressed by the noise threshold. This is because at these energies, it is more likely that the energy shared with an adjacent pixel is below the noise threshold, resulting in a failure to identify true shared events. As the energy increases and the noise threshold is less likely to suppress a shared event, the shared events proportion begins to level off. This occurs at ∼23 keV where the shared events proportion is ∼35%. However, at incoming photon energies above the K-edge of high-Z atoms (labelled in Figure 9), fluorescence photon generation results in a significant increase of shared events. Another factor which results in a further increase of shared events is the jump in attenuation at the absorption edges. This means the electron charge cloud will on average need to drift further to reach the anode and increase in size—resulting in a higher probability of inducing charge in multiple pixels. Above the K-edge absorption energies of the Cd and Te atoms, the shared events proportion begins to level-off again at ∼35 keV (46% shared events—as predicted by the model). The number of shared events then gradually rises to 67% at 140.5 keV as the charge cloud size increases with photon energy.

The charge cloud size is dependent on the bias voltage applied across the detector as this determines the electric field strength under which the charges drift [38]. At higher bias voltages, less charge sharing will occur as charge carriers drift to the electrodes more quickly allowing less time for the charges to spread due thermal diffusion and carrier repulsion. However, the trapping of carriers during drift leads to a build-up of charge which form internal electric fields that act against the externally applied voltage [42]. This polarization phenomenon results in a net effective field strength that is weaker than the desired field strength expected from the applied bias—a consequence of this is an increase in charge sharing proportions. For an applied voltage of −500 V, 1 mm Schottky contact CdTe has been shown to have an effective voltage across the detector of −425 V [5]. Consequently, when simulating the CdTe detector in [26], the model required the use of the weaker effective bias in order to correctly predict the experimental charge sharing rates. In contrast, the excellent agreement between the charge sharing rates predicted by the detector model and the experimental values for the HF-CdZnTe, were obtained through simulations at the externally applied detector bias of −750 V. This suggests that the electric field strength across the HF-CdZnTe detector is more uniform and not degraded by polarization effects. This will likely be due to a combination of the better hole carrier lifetime-mobility product in the HF-CdZnTe and the different contact technology compared to the Acrorad Schottky contacts.

### 3.4. True Charge Sharing Proportions

**Figure 10:**
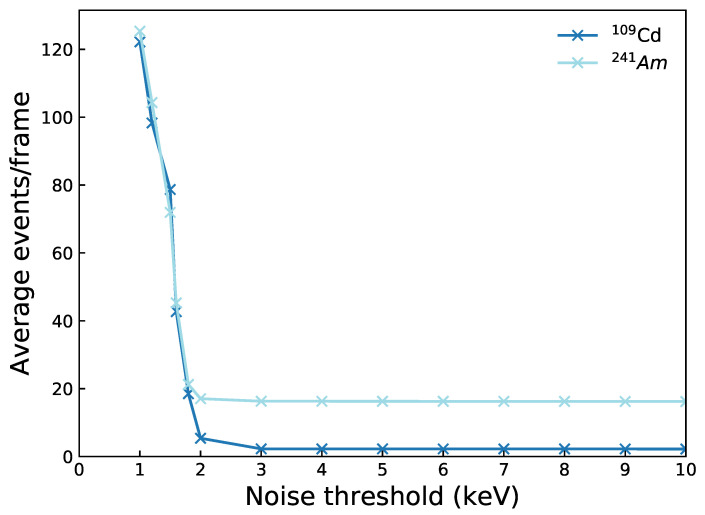
Average number of all event types per frame as a function of the noise threshold for the ${}^{109}$Cd and ${}^{241}$Am observations with the HF-CdZnTe detector.

In Section 3.3, the suppression of shared events due to the noise threshold was discussed. In this section we estimate the true charge sharing proportions when no noise threshold is applied by using simulations from the detector model. Very low energies recorded by a detector will be dominated by noise events due thermal charge leakage. It is therefore impossible to experimentally measure the number of shared events at a threshold of 0 keV as multi-pixel events due to charge sharing or noise can not be distinguished. For the HF-CdZnTe detector at 28 ${}^{\circ }$C, a minimum noise threshold of 2 keV is required to suppress the majority of noise counts. This can be seen by Figure 10 which shows the average number of events per frame (including all event types) as a function of the noise threshold for the ${}^{109}$Cd and ${}^{241}$Am HF-CdZnTe observations. Above a 2 keV threshold the average events per frame vary little, indicating that the majority of events are due to absorbed photons and not noise. The detector model does not simulate thermal or electronic noise events, meaning shared events can be estimated at a 0 keV threshold.

**Figure 11:**
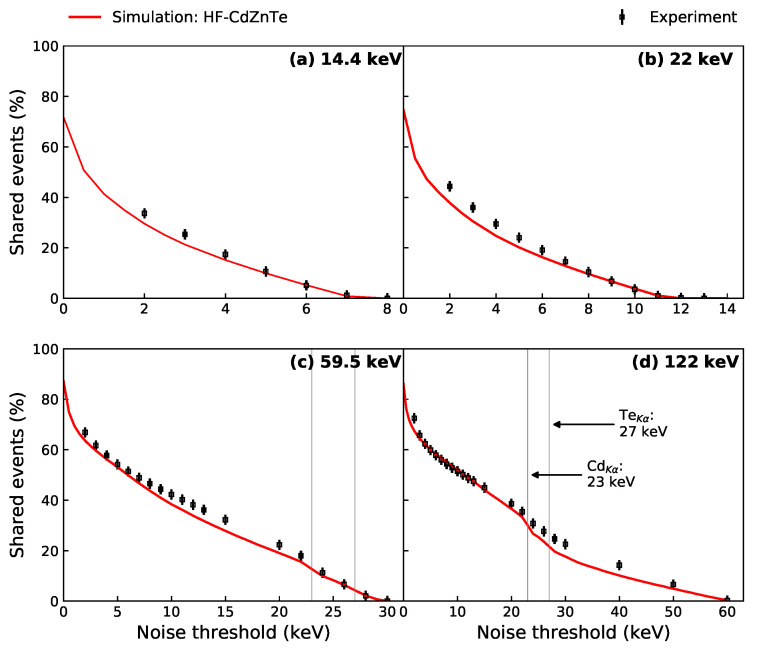
Proportion of charge sharing as a function of the applied noise threshold for the (**a**) 14.4 keV, (**b**) 22 keV, (**c**) 59.5 keV and (**d**) 122 keV photopeaks, comparing experiment and simulation. The noise thresholds used on the experimental data are shown by the markers. For the simulated data, noise thresholds were applied at 1 keV intervals. The errors on the experimental values are estimated at ±2% from varying the energy window around the photopeak between ±10–15%. K-shell fluorescence energies are only labelled once in panel (**d**), but indicated by the solid vertical lines in both (**c**,**d**).

Figure 11 shows the proportion of shared events as a function of the noise threshold at four different photopeak energies, for both the HF-CdZnTe experimental data and simulated data. The proportions were again calculated using 10% energy windows around the photopeaks, for every applied threshold. For the experimental data, the analysis was done down to the lowest threshold at which the number of noise events was negligible (2 keV). The largest threshold used depended on the photopeak energy. At a noise threshold of half the photopeak energy, the shared events proportion is expected to go to zero since the threshold will be equal to or greater than the maximum amount of energy that can be shared or remains in a single pixel. The charge sharing predictions from the model show good agreement with the experimental values. As larger noise thresholds are applied, the number of shared events decreases exponentially. For the 59.5 and 122 keV photopeaks (energies above the material absorption edges), the exponential decrease is interrupted by sudden drops at the fluorescence photon energies (i.e., at the first simulated point above the fluorescence energy).

**Figure 12:**
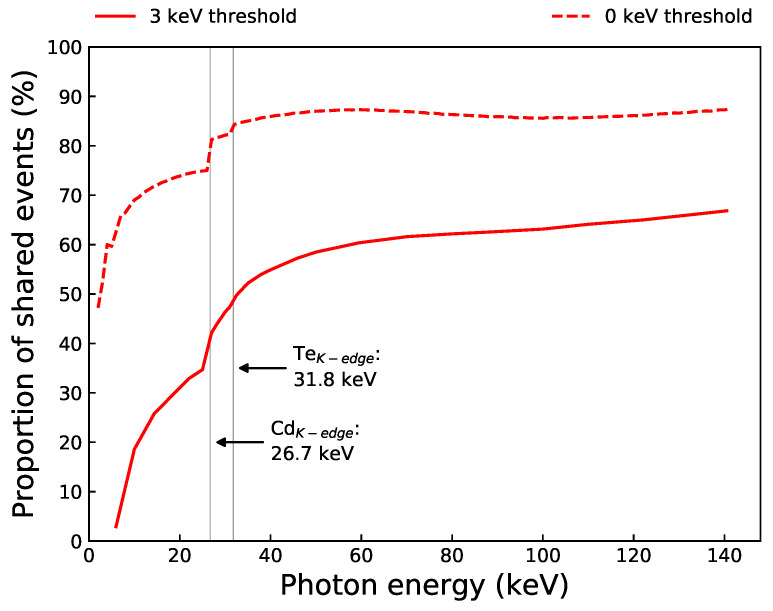
Proportion of charge sharing as a function of photon energy, calculated from simulated data from the Monte-Carlo detector model. Shown for two different noise thresholds. The 3 keV simulated curve is from Figure 9, shown for comparison.

Figure 12 shows the proportion of shared events as a function of incoming photon energy when no noise threshold is applied, compared with the simulated result from Figure 9 when a 3 keV threshold was used. The 0 keV threshold curve shows the true percentage of events that are shared in the HF-CdZnTe detector, which is significantly larger than when a 3 keV threshold is applied. At a photon energy of 5.95 keV, the true amount of charge sharing is 63%, compared to 3.0% when a 3 keV threshold was used. The number of true shared events increases rapidly at very low photon energies as more charge carriers are excited by the absorbed photon. At 122 keV, 86% of all events exhibit some charge sharing in the HF-CdZnTe detector. Although this is a very large proportion, 21% of those shared events are sharing 3 keV or less.

## 4. Discussion

Charge sharing in small-pixel Cd(Zn)Te detectors is significant and is expected to increase with detector thickness (Figure 9). Even for soft X-rays, simulations of the HF-CdZnTe detector with no thermal or electronic noise, revealed that the majority of events still exhibit some charge sharing (Figure 12). The true charge sharing proportions, particularly at lower photon energies, is suppressed by the low-noise threshold typically applied to detector recorded counts during post-processing to remove noise. For applications such as in astronomy where each count is vital to achieve good signal-to-noise statistics, and good spectral resolution is necessary to identify emission lines, large proportions of charge sharing become an issue when the detector’s spectral response to shared events suffers due to incomplete charge collection.

The HF-CdZnTe detector showed significantly improved energy resolution for the charge sharing photopeaks over the CdTe detector (Figure 7a). The better spectral response of shared events in the HF-CdZnTe detector is due to significantly less charge loss across the inter-pixel gap, revealed by the energy split analysis for the adjacent bipixels (Figure 3) and quantified by determining the charge loss contribution to photopeak broadening, $\sigma_{c}$ (Figure 7b).

The reduced charge loss in shared events for the HF-CdZnTe detector is expected to primarily be due to the smaller 25 μm inter-pixel spacing (compared with 50 μm in the CdTe detector), resulting in improved charge collection in regions close to the pixel gaps. Through simulation it has been shown that the electric field lines become distorted within the inter-pixel spacing close to the anode and have a weaker field strength [43,44]. Carriers drifting in these regions are therefore more likely to become trapped and stop inducing a signal [18], resulting in charge loss. Smaller inter-pixel gaps are thought to minimise this effect as electric field distortion is less pronounced and fewer charges traverse inter-pixel regions. This is in line with our findings which show less charge loss for the detector with smaller pixel gaps (i.e., smaller pixel gap to pixel pitch ratio)—the HF-CdZnTe detector. However, given the number of differences between the two detectors (electrode technology, thickness, compound material), we can not confidently make this link based on our comparison—where the CdTe serves instead as a reference point to compare the HF-CdZnTe charge sharing performance with.

The strong correlation between $\sigma_{c}$ and interaction depth (Figure 8b) suggests that hole carrier trapping influences the severity of charge loss in charge sharing events. At lower photon energies, due to shallower attenuation depths, the poor hole transport has a smaller impact on the energy resolution of shared events than at larger energies. The correlation of charge loss with depth of interaction due to the poor hole transport in high-Z semiconductors has also been reported in other works [45,46]. The lower rate of $\sigma_{c}$ with interaction depth (Figure 8b) in the HF-CdZnTe over the CdTe might be a benefit of the improved hole transport, although a better comparison with standard CdZnTe material is required to confidently determine this. Abbene et al. [16], who compared charge sharing and charge loss in 3 × 3 pixelated array detectors using 2 mm thick Redlen HF-CdZnTe material with 500 μm and 250 μm pixel pitch, both with 50 μm pixels gaps, found that charge loss for 59.5 keV photons worsened when the ratio between pixel gap and pixel pitch increased. Therefore, despite the improved hole transport in HF-CdZnTe, charge loss is predominately influenced by the pixel gap and pixel pitch size.

In detectors that can determine the interaction depth of events, through ASIC pixels capable of measuring both positive and negative polarity signals or otherwise, the correlation between the charge loss in shared events and interaction depth can be tested more vigorously. Furthermore, in such a detector, discrimination techniques could be used to remove shared events with interaction positions close to the anode. This could further improve the energy resolution of high energy shared events, although at the cost of losing counts. Depth of interaction correction techniques could also be applied, and has already been done successfully to correct for charge loss and improve spectral performance in similar detectors [16,45].

The spectroscopic performance of the HF-CdZnTe detector compares favourably to the performance achieved using spectroscopic-grade CdZnTe from Redlen technologies [47], also coupled with the HEXITEC ASIC in a detector of 2 mm thickness and 250 μm pixel pitch. Using the spectroscopic-grade CdZnTe, the average FWHM of the 59.5 keV photopeak for the entire detector was 1.74 ± 0.39 keV for isolated events only, compared with 0.95 ± 0.07 keV achieved with the HF-CdZnTe detector here. Veale et al. [47] showed evidence of local variations in the electric field, linked to the build up of space charge due to carrier trapping and the use of blocking contacts [48], which would damage spectroscopic performance. The agreement between the experimentally determined charge sharing rates and simulation set to the applied external bias of −750 V (Figure 9), suggests that the electric field strength within the HF-CdZnTe does not significantly suffer from local distortions and is uniform. Little spatial variation in the charge sharing proportions across the HF-CdZnTe detector support this finding [28]. This is a likely explanation for the better isolated event photopeak performance of the HF-CdZnTe compared with the spectroscopic grade CdZnTe. The lack of local variations in the electric field is also evidence of better hole transport, as the improved electric field uniformity will be related to fewer carriers trapping.

Thomas et al. [24] reported a reduced electron mobility-lifetime product in the HF-CdZnTe at the cost of the improved hole transport. For the 2 mm detector thickness, we have not observed any indication that this has limited the spectroscopic performance. Whether the reduced electron transport will begin to limit performance in thicker HF-CdZnTe detectors, remains to be investigated.

It should be noted that the results presented in this work were calculated from whole-detector spectra (i.e., the spectrum recorded by each pixel summed). Performance will vary slightly with each pixel due changes in defect concentration and purity across a crystal. Therefore, results on the charge sharing proportion and charge loss contribution for example, reflect an average performance per pixel. This is also a source of uncertainty and leads to some of the large errors, which capture the pixel per pixel variation. To reduce uncertainties and obtain limits on the best and worst possible performance of the materials, further analysis on a per pixel basis is required.

## 5. Conclusions

Developed for high-flux applications to overcome the polarization phenomenon, Redlen technologies have grown high-flux capable CdZnTe sensor material (HF-CdZnTe) with improved hole carrier transport. We have shown that 2 mm thick HF-CdZnTe used in a detector with 250 μm pixel pitch and 25 μm pixel gaps, results in a detector that exhibits excellent spectral performance at room-temperature. For single pixel events, the achieved energy resolution is similar to that of the best performing CdTe detectors. Charge sharing events however show the biggest improvement, with much smaller energy resolutions due to better charge collection across the inter-pixel regions.

We discussed that the smaller 25 μm pixel gaps are likely the primary factor behind the reduced charge loss to the inter-pixel regions. However, evidence was shown that links the poor hole transport in Cd(Zn)Te with the severity of charge loss in multi-pixel events as a function of photon energy, due to depth of interaction effects. Comparison between experimental and simulated charge sharing rates suggest a uniform electric field profile in the HF-CdZnTe detector, believed to be related to the better hole transport. Given the significant proportions of charge sharing measured in small pixel Cd(Zn)Te detectors, which has been shown, a good spectral response for shared events is vital to maximise the number of counts that can be measured with a good energy resolution.

It was also shown that some shared events not affected by charge loss due originating from sensor emitted fluorescence, can be recovered with good spectral resolution by including diagonally adjoined events. Whether more counts are recovered or lost by including diagonally adjoined events was shown to depend on the detector recorded frame occupancy, given as a threshold. This threshold could be determined using the detector Monte-Carlo model which accurately predicted the photon pileup and fluorescence incidence rates of diagonal bipixels.

The HF-CdZnTe detectors show potential for applications which require small pixels for good spatial resolution, while maintaining good energy resolution at hard X-ray energies.

## Figures and Tables

**Table 1:** Different radioisotope sources used to acquire flood images with the HF-CdZnTe detector. One flood acquisition was taken with each source, with the ASIC frame read out set to 1.6 kHz. Prominent photopeak energies used for calibration and charge sharing analysis obtained from each source are shown. A nominal uncertainty of ±10 eV is used for the photopeaks unless the photopeak energy is made up of multiple unresolved peaks (i.e., K${}_{\alpha 1}$ and K${}_{\alpha 2}$ emissions). In this case the error is estimated taking into account the relative intensity of each peak making up the photopeak.

Radioisotope Source	Photopeak Energies (keV)	Frame Occupancy (%)
${}^{55}$Fe	5.95 ± 0.01	0.4
${}^{109}$Cd	22.00 ± 0.10	0.2
	24.90 ± 0.01	
	88.00 ± 0.01	
${}^{241}$Am	59.54 ± 0.01	0.4
${}^{57}$Co	14.40 ± 0.10	0.3
	122.10 ± 0.05	
	136.50 ± 0.10	
${}^{99m}$Tc	18.30 ± 0.10	0.1
	140.50 ± 0.10	

**Table 2:** List of ${}^{241}$Am flood images taken with the HF-CdZnTe detector. Different source distances and ASIC frame rates were used to obtain a range of frame occupancy rates in the data. ${}^{a}$This is the flood observation also used for calibration, listed in Table 1.

Source Distance (cm)	Frame Rate (Hz)	Photon Flux (·10${}^{3}$ ph s${}^{-1}$ mm${}^{-2}$)	Frame Occupancy (%)
38 ${}^{a}$	1600	0.2	0.4
22	1600	0.6	1.3
18.5	1600	0.8	2.2
13.5	1600	1.5	4.0
11	1600	2.3	5.9
7	1600	5.5	13.2
6.5	1600	6.4	13.9
5	1600	10.7	20.1
3	1600	27.8	31.1
7	560	5.5	32.8
7	440	5.5	39.5
7	378	5.5	44.7
7	312	5.5	51.1

**Table 3:** Fitting results of Equation (3) in Figure 7a. The fit parameters correspond to the standard deviation due to electron noise $\sigma_{a}$ and incomplete charge collection $\sigma_{c}$. For isolated events, it was assumed $\sigma_{c}=0$. For multi-pixel events, it was assumed $\sigma_{a}$ is equal to the value obtained for the isolated events of the respective detector. No fit was found for the CdTe bipixel and quadpixel FWHM values. The errors are standard errors obtained from the least-squares fit of Equation (3).

	Isolated (m=1)	Bipixel (m=2)	Quadpixel (m=4)
	$\sigma_{a}$ (keV)	$\sigma_{c}$ (keV)	$\sigma_{c}$ (keV)
CdTe	0.36 ± 0.03	-	-
HF-CdZnTe	0.37 ± 0.03	0.41 ± 0.12	0.64 ± 0.16

## Data Availability

Data available on request.

## Abbreviations

The following abbreviations are used in this manuscript:

STFC	Science and Technology Facilities Council
HEXITEC	High Energy X-ray Imaging Technology
ASIC	Application Specific Integrated Circuit
DAQ	Data acquisitions system
XRF	X-ray fluorescence
CdTe	Cadmium telluride
HF-CdZnTe	High-flux cadmium zinc telluride
Cd(Zn)Te	CdTe and CdZnTe
ADU	Analogue-to-digital unit
FWHM	Full width at half maximum
LHS	Left hand side
